# Blood Levels of Monoamine Precursors and Smoking in Patients with Schizophrenia

**DOI:** 10.3389/fpubh.2016.00182

**Published:** 2016-08-30

**Authors:** Ashwin Jacob Mathai, Jyoti Kanwar, Olaoluwa Okusaga, Dietmar Fuchs, Christopher A. Lowry, Xiaoqing Peng, Ina Giegling, Annette M. Hartmann, Bettina Konte, Marion Friedl, Claudia Gragnoli, Gloria M. Reeves, Maureen W. Groer, Richard N. Rosenthal, Dan Rujescu, Teodor T. Postolache

**Affiliations:** ^1^Mood and Anxiety Program, University of Maryland School of Medicine, Baltimore, MD, USA; ^2^Saint Elizabeths Hospital Psychiatry Residency Training Program, Washington, DC, USA; ^3^Department of Psychiatry and Behavioral Sciences, The University of Texas Health Science Center at Houston, Houston, TX, USA; ^4^Division of Biological Chemistry, Biocenter Innsbruck Medical University, Innsbruck, Austria; ^5^Department of Integrative Physiology, Center for Neuroscience, University of Colorado Boulder, Boulder, CO, USA; ^6^Department of Psychiatry, Martin-Luther-University of Halle-Wittenberg, Halle, Germany; ^7^Division of Endocrinology, Sidney Kimmel Medical College at Thomas Jefferson University, Philadelphia, PA, USA; ^8^Public Health Sciences, Penn State College of Medicine, Hershey, PA, USA; ^9^Division of Child and Adolescent Psychiatry, University of Maryland School of Medicine, Baltimore, MD, USA; ^10^University of Maryland Child and Adolescent Mental Health Innovations Center, Baltimore, MD, USA; ^11^University of South Florida, Tampa, FL, USA; ^12^Department of Psychiatry, Icahn School of Medicine at Mount Sinai, New York, NY, USA; ^13^VISN 5 Capitol Health Care Network Mental Illness Research Education and Clinical Center (MIRECC), Baltimore, MD, USA; ^14^Rocky Mountain MIRECC, Denver, CO, USA

**Keywords:** phenylalanine, tyrosine, kynurenine, tryptophan, schizophrenia, smoking

## Abstract

Smoking is highly prevalent in patients with schizophrenia and exerts a negative impact on cardiovascular mortality in these patients. Smoking has complex interactions with monoamine metabolism through the ability of cigarette smoke to suppress Type 1 T helper cell (Th1) type immunity, the immunophenotype that is implicated in phenylalanine hydroxylase (PAH) dysfunction and tryptophan (Trp) breakdown to kynurenine (Kyn) *via* indoleamine 2,3-dioxygenase. Nicotine also induces tyrosine hydroxylase (*TH*) gene expression, leading to increased synthesis of catecholamines. Furthermore, there is evidence for PAH dysfunction in schizophrenia. This study aimed to compare the plasma levels of selected monoamine precursors and their metabolites in smokers vs. non-smokers in a large sample of patients with schizophrenia. We measured plasma phenylalanine (Phe), tyrosine (Tyr), Trp, and Kyn levels using high-performance liquid chromatography and calculated Phe:Tyr and Kyn:Trp ratios in 920 patients with schizophrenia. Analysis of variance and linear regression analyses were used to compare these endpoints between three groups of patients with schizophrenia: (1) current smokers, (2) past smokers, and (3) non-smokers. There were significant differences among the three groups with regards to Tyr levels [*F*_(2,789)_ = 3.77, *p* = 0.02], with current smokers having lower Tyr levels when compared with non-smokers (*p* = 0.02). Kyn levels and Kyn:Trp ratio were different among the three groups [*F*_(2,738)_ = 3.17, *p* = 0.04, *F*_(2,738)_ = 3.61, *p* = 0.03] with current smokers having lower Kyn levels (*p* = 0.04) and higher Kyn:Trp ratio (*p* = 0.02) when compared with past smokers. These findings need to be replicated with protocols that include healthy controls to further elucidate the neurobiological underpinnings of altered Tyr and Kyn levels in smokers. Results do suggest potential molecular links between schizophrenia and smoking that may represent biomarkers and treatment targets for reducing an important modifiable cause of general morbidity and mortality in patients with schizophrenia.

## Introduction

Cigarette smoking is more prevalent in patients with schizophrenia in comparison to the general population as well as patients with other severe mental illnesses (1). Smoking has recently been associated with increased risk of developing schizophrenia (2), and it may also represent an expression of general vulnerability for substance use disorders in schizophrenia (3). Smoking is also an important modifiable risk factor for increased cardiovascular morbidity and mortality reported in patients with schizophrenia (4, 5). Furthermore, smokers with schizophrenia appear to be at an increased risk for suicide (6, 7) when compared with non-smokers with the illness. Recognizing and addressing smoking as a major comorbidity in patients with mental illness, especially schizophrenia, is yet to gain impetus in clinical practice (8).

Smoking leads to increased dopaminergic neurotransmission (9) and is purported (10) to improve “hypofrontality,” which is manifested as negative and cognitive symptoms of schizophrenia (11). Cigarette smoke has an inhibitory effect on monoamine oxidase, which may also increase the availability of dopamine due to its reduced metabolism (12). Impairments in attentional processes and other cognitive functions in schizophrenia are linked to abnormal sensory gating and dysfunction of alpha-7 nicotinic cholinergic receptor, which are characterized by a deficit in P50 auditory evoked response (10) and prepulse inhibition (PPI) (13). Smoking briefly normalizes P50 amplitude in schizophrenia probands and their relatives, and nicotine improves PPI and cognition in patients with schizophrenia (14).These findings lend evidence to the self-medication hypothesis which suggests that increased smoking in schizophrenia may be an attempt to compensate for attentional deficits and other symptoms of the illness (15).

Smoking may also reduce some side effects of antipsychotic medications. For example, polycyclic aromatic hydrocarbons in cigarette smoke induce cytP4501A2, which increases metabolism of medications (16), thus reducing side effects of these medications. Increased prevalence of smoking in schizophrenia is also proposed to be due to an alleviation of extrapyramidal side effects of neuroleptics (17), a finding that is aligned with the evidence for a protective effect of smoking in Parkinson’s disease, likely due to the ability of nicotine to improve dopaminergic neurotransmission in the striatum (17). It is therefore possible that patients with schizophrenia smoke to alleviate side effects of antipsychotic medications.

Phenylalanine (Phe) is an essential amino acid involved in the synthesis of dopamine, the neurotransmitter most commonly implicated in the pathogenesis of schizophrenia. Phenylalanine hydroxylase (PAH) converts Phe to tyrosine (Tyr). Tyr is further converted into dopamine *via* a two-step enzymatic reaction involving Tyr hydroxylase (rate-limiting enzymatic reaction of catecholamine synthesis) and aromatic l-type amino acid decarboxylase (18, 19). Phe:Tyr ratio is considered to be an estimate of PAH activity (20). The PAH activity is modulated by (6R)-l-erythro-5,6,7,8-tetrahydrobiopterin (BH4), an essential cofactor for aromatic amino acid hydroxylases including PAH (21). Type 1 T helper (Th1) cell immune activation leads to release of pro-inflammatory cytokines and decreased activity of PAH despite stimulated BH4 production (22), thus decreasing Phe turnover and increasing Phe:Tyr ratio. PAH dysfunction during activation of Th1-type immunity is thought to be mediated by oxidative stress-induced depletion of BH4 in the context of a pro-inflammatory state (20). Moreover, Th1 immune activation leads to shunting of tryptophan (Trp) away from serotonin synthesis toward kynurenine (Kyn) production *via* the induction of indoleamine 2,3-dioxygenase (IDO) (23) by Th1 cytokines like interferon gamma (IFNγ). Kyn:Trp is considered a sensitive indicator of IDO activity and Trp degradation; an elevated ratio signifying dominant Th1 immune activation (24).

Elevated plasma Phe and reduced Tyr levels seen in patients with schizophrenia are presumable consequences of an inflammation-mediated decrease in PAH activity (25–28). High Phe levels lead to increased levels of Phe in the brain, which in turn decreases the activity of aromatic amino acid hydroxylases including tyrosine hydroxylase (TH) and Trp hydroxylase, thus decreasing overall monoamine synthesis in the brain (18). Abnormal Phe kinetics was demonstrated in patients with schizophrenia using radiolabeled Phe breath tests (29). PAH gene polymorphisms have been identified in schizophrenia (30, 31).

**Figure 1:**
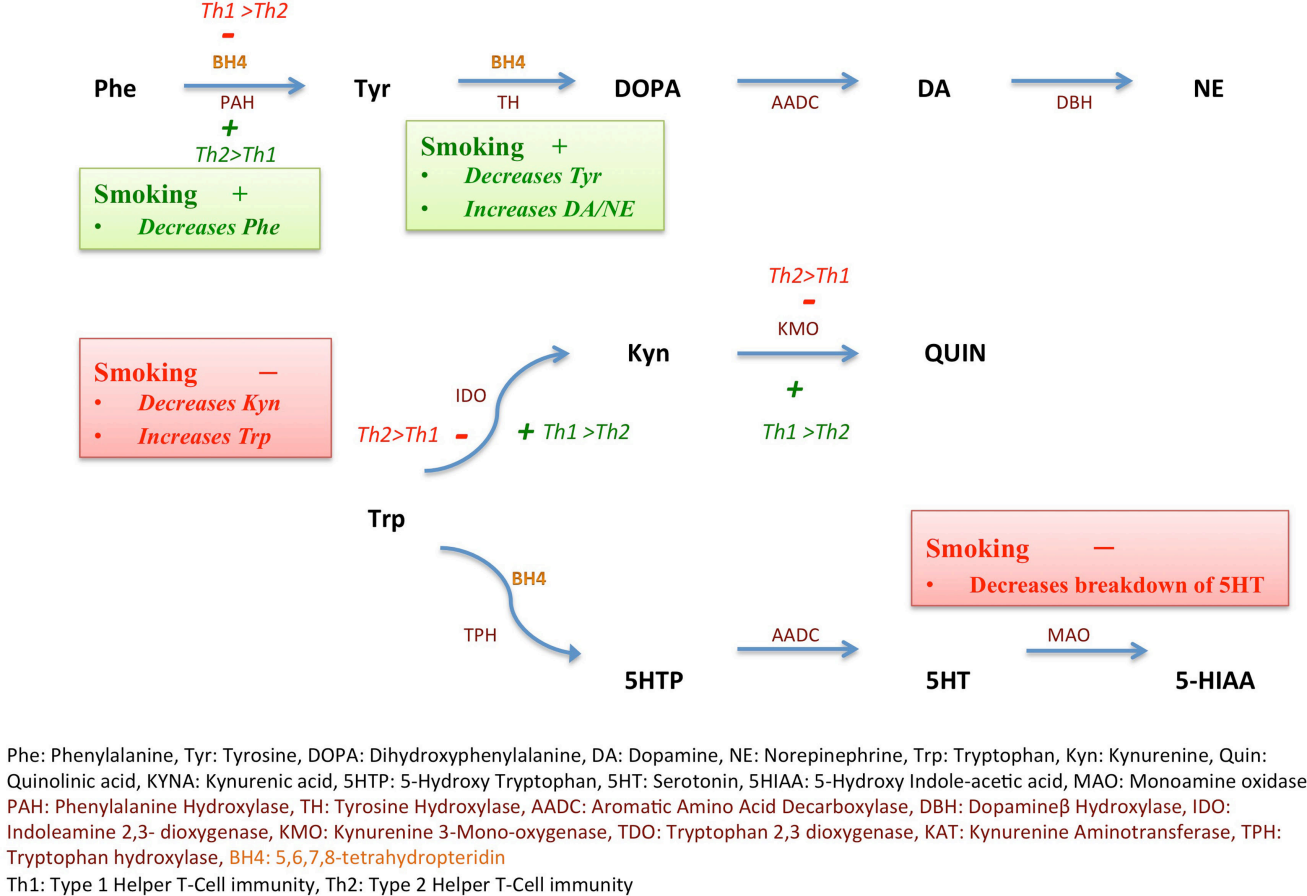
**Inflammation, smoking, and monoamine metabolism**. Nicotine increases the expression of tyrosine hydroxylase (*TH*) gene as depicted in green (+), thus decreasing tyrosine (Tyr) levels and increasing downstream catecholamine synthesis. Smoking also favors Type 2 helper cell (Th2) immunity over Type 1 helper cell (Th1) immunity, in turn, decreasing the activity, depicted in red (−), of indoleamine 2,3-dioxygenase (IDO), consequently maintaining tryptophan (Trp) levels and decreasing kynurenine (Kyn). Th2 immune activation favored by smoking also increases the activity of phenylalanine hydroxylase (PAH), depicted in green (+). Smoking inhibits monoamine oxidase enzyme, depicted in red (−) and decreases breakdown of serotonin (5-hydroxytryptamine, 5-HT) into 5-hydroxyindoleacetic acid (5-HIAA). Th1 immunity *via* interferon gamma (IFNγ) triggers reactive oxygen species (ROS)-induced depletion of tetrahydrobiopterin (BH4) leading to PAH dysfunction.

Cigarette smoking is known to favor Th2 immune responses while suppressing Th1 immunity (32). Smoking decreases the activity of IDO by inhibiting Th1 responses *via* suppression of IFNγ, a major Th1 cytokine, thereby increasing Trp levels and decreasing Kyn:Trp ratio (32) (Figure 1). On the other hand, smoking may increase the activity of PAH *via* carbon monoxide (CO)-mediated suppression of IFNγ (22, 33), thus decreasing Phe:Tyr ratio.

Alternatively, nicotine reliably increases the expression of *TH* gene, protein, and TH activity both centrally (34) and in adrenal medulla (35, 36), possibly leading to decreased Tyr levels. A sustained increase in TH activity after a single administration of nicotine (37) has been described. Unsurprisingly polymorphisms in the *TH* gene are linked to susceptibility to smoking (38, 39).

The unusually high rate of smoking in patients with schizophrenia and disproportionately high mortality rate from heart disease in this population demands scrutiny of the complex associations of smoking and schizophrenia. We have previously reported that Phe levels and Phe:Tyr ratio were elevated in the same cohort of patients with schizophrenia when compared with controls (40). To our knowledge, there have been no studies investigating links between blood levels of molecular precursors of dopamine and serotonin in relationship to smoking in patients with schizophrenia.

## Materials and Methods

The local ethics committee of Ludwig Maximilians University, Munich, Germany approved the study, and the additional analysis of the data at the University of Maryland, Baltimore, MD, USA was determined as exempt by the Institutional Review Board of the University of Maryland School of Medicine, Baltimore, MD, USA. Written consent was obtained from all study participants after detailed description of study procedures. An experienced clinical psychiatrist evaluated the participants’ capacity to consent to participate in the study.

### Participants

Nine hundred and twenty patients with a diagnosis of schizophrenia were enrolled from inpatient and outpatient settings in the Munich area in Germany. Structured Clinical Interview for DSM-IV TR Axis I Disorders, Research Version, Patient Edition (SCID) was used to confirm the diagnosis of schizophrenia in patients (41). Subjects who met criteria for schizoaffective disorder, schizophreniform disorder, substance-induced psychosis, and psychotic disorder not otherwise specified (NOS) were excluded. Positive and negative syndrome scale (PANSS) was used to measure severity of symptoms in all patients (42). According to their answers on the Fagerstrom Nicotine Dependence Test (43), the patients were divided into three categories: (1) non-smoker – those who smoked less than 100 cigarettes in their lifetime (*n* = 239); (2) past smoker – those who smoked more than 100 cigarettes over their lifetime, but no cigarettes in the past week (*n* = 130); and (3) current smoker – who smoked more than 100 cigarettes over their lifetime and any amount in the past week (*n* = 551). Antipsychotic medications were recorded, and the chlorpromazine (CPZ) equivalent of antipsychotic dosage was calculated.

### Measurement of Plasma Phenylalanine, Tyrosine, Kynurenine, and Tryptophan

No dietary restrictions or fasting protocols were imposed. Blood samples were obtained from a forearm vein and drawn in EDTA-containing tubes. After centrifuging the samples for 10 min at 4°C, the resulting plasma was aliquoted into Eppendorf tubes, which were frozen immediately at −80°C. Until analysis, the samples were frozen at −80°C. Using high-performance liquid chromatography (HPLC), Phe, Tyr, Kyn, and Trp levels were determined by monitoring their natural fluorescence as described elsewhere (44, 45). Peak height counts were employed to determine concentrations after referring Phe, Tyr, Trp, and Kyn to internal standard, 3-nitro-l-tyrosine.

### Statistical Analysis

The ratio between plasma Phe and Tyr was used to estimate the activity of the enzyme PAH (24). Kyn:Trp ratio was used to determine the activity of IDO. As the distributions of Phe, Tyr, Kyn, Trp, Phe:Tyr, and Kyn:Trp ratio were skewed, we applied logarithmic transformation to ensure normal distribution. One-way analysis of variance (ANOVA) was conducted to determine whether there were any differences in Phe, Tyr, Kyn, Trp, Phe:Tyr, and Kyn:Trp levels between current smokers, past smokers, and non-smokers, and Tukey’s *post hoc* test was used after detecting significant differences. To assess smoking as a trait, current and past smokers were combined into a single group and compared with non-smokers. Logistic regression models were built to estimate associations between smoking status (smoker vs. non-smoker) as dependent variable and as independent variables, in succession: (a) Tyr, Phe, and their ratio and (b) Kyn, Trp, and their ratio, with age, sex, BMI, and CPZ equivalent as covariates. We also used linear regressions to estimate associations between PANNS symptoms scores and Tyr, Phe, and their ratio and Kyn, Trp, and their ratio with successive adjustment for the above listed covariates.

## Results

### Sample Demographic and Clinical Characteristics

Age differed among the three groups of patients with current smokers on average being younger than past smokers (*p* < 0.001) and non-smokers as a group (Table 1). Current smokers had statistically higher scores on the PANSS-positive symptoms subscale (*p* = 0.004). There was a significant difference between the three groups with regards to PANSS-positive symptoms subscale score after controlling for age, gender, and BMI [*F*_(2,886)_ = 5.75, *p* = 0.003]. On *post hoc* analysis, current smokers had higher PANSS-positive subscale scores when compared with non-smokers (*p* = 0.007). The difference in positive symptoms subscale between current smokers and past smokers was trending toward significance (*p* = 0.06). Data on smoking status and Phe/Tyr and Kyn/Trp levels were available for 844 and 796 patients, respectively.

**Table 1:** **Sample demographic and clinical characteristics**.

Variable	Current smokers (*n* = 551)	Past smokers (*n* = 130)	Non-smokers (*n* = 239)	*p*-Value ANOVA
Age, years, mean (SD)	36.6 (11.2)	39.8 (11.8)	40 (11.8)	<0.0001
Gender, male, *n* (%)	389 (70)	75 (57)	120 (50)	
BMI mean (SD)	27.1 (5.5)	27 (5.6)	27.1 (5.2)	0.9
CPZ equivalent mean (SD)	542 (1,545)	477 (935)	350 (382)	0.15
PANSS mean (SD)				
Positive symptoms mean (SD)	28.2 (6.3)	26.8 (6.5)	26.8 (6.2)	0.004
Negative symptoms mean (SD)	24.7 (6.8)	23.4 (9)	24.1 (7.8)	0.175
General psychopathology mean (SD)	49.3 (11.1)	48.6 (13.6)	49.6 (11.7)	0.729

*BMI, body mass index; CPZ equivalent, chlorpromazine equivalent; PANSS, positive and negative syndrome scale*.

### Smoking Status and Phe, Tyr, and Phe:Tyr Ratio

There was a significant difference in Tyr levels between the three groups (current smoker, past smoker, and non-smoker) [*F*_(2,841)_ = 6.48, *p* = 0.001] on crude analysis with current smokers having lower and non-smokers having higher Tyr levels. The effect persisted after controlling for age, sex, BMI, and CPZ equivalent dosage [*F*_(2,789)_ = 3.77, *p* = 0.02] (Table 2). There were no differences among the three groups in Phe levels [*F*_(2,789)_ = 0.76, *p* = 0.47] and Phe:Tyr ratio [*F*_(2,789)_ = 1.34, *p* = 0.26]. On *post hoc* pairwise analysis of the difference between the three groups in Tyr levels, current smokers had lower Tyr levels when compared with non-smokers (*p* = 0.006) and past smokers (*p* = 0.02), but there was no difference between past smokers and non-smokers (*p* = 0.99). This difference persisted after controlling for age, sex, BMI, and PANSS-positive symptoms between current smokers and non-smokers (*p* = 0.02) and was marginally trending toward significance for difference between current smokers and past smokers (*p* = 0.05).

**Table 2:** **Means and SDs of monoamine precursors/metabolites and differences among the three groups after log transforming levels and adjusting for age, sex, BMI, and CPZ equivalent**.

Precursor/metabolite (units)	Current smoker mean ± SD (*n*)	Past smoker mean ± SD (*n*)	Never smoker mean ± SD (*n*)	ANOVA *F* ratio/*p*-Value
Phe (μmol/L)	75.11 ± 36.02 (506)	80.715 ± 37.64 (119)	76.53 ± 33.45 (219)	0.75/0.47
Tyr (μmol/L)	74.185 ± 32.89 (506)	86.68 ± 50.19 (119)	82.71 ± 35.92 (219)	**3.77/0.02**
Phe:Tyr	1.14 ± 0.64 (506)	1.08 ± 0.54 (119)	1.06 ± 0.66 (219)	1.33/0.26
Kyn (μmol/L)	2.60 ± 1.47 (470)	3.07 ± 1.85 (113)	2.81 ± 1.63 (213)	**3.17/0.04**
Trp (μmol/L)	65.40 ± 52.82 (470)	63.97 ± 42.21 (113)	61.30 ± 16.52 (213)	0.41/0.66
Kyn:Trp	44.44 ± 26.96 (470)	54.92 ± 39.86 (113)	48.94 ± 36.64 (213)	**4.07/0.02**

*Phe, phenylalanine; Tyr, tyrosine; Phe:Tyr, phenylalanine:tyrosine; Kyn, kynurenine; Trp, tryptophan; Kyn:Trp, kynurenine:tryptophan; Phe:Tyr, phenylalanine:tyrosine. Bold font numerals denote statistically significant results*.

Smoking as a trait was assessed by consolidating current and past smokers into one group (*n* = 605) and comparing them to non-smokers (*n* = 213) after including age, sex, BMI, and PANSS-positive symptoms subscale scores in a logistic regression model. In this multivariate model, smoking (current smoking and past smoking) was associated with lower Tyr levels when compared with non-smoking (OR = 2.37, CI = 1.107–5.090, *p* = 0.03). After further including CPZ equivalent dosage in the model, the relationship was trending toward significance (*p* = 0.06). There was no significant association with Phe:Tyr ratio (*p* = 0.06) or Phe (*p* = 0.70) levels.

### Smoking Status and Trp, Kyn, and Kyn:Trp Ratio

There were statistically significant differences in Kyn levels among the three groups (current smoker, past smoker, and non-smoker) after adjusting for age, sex, BMI, and CPZ equivalent [*F*_(2,738)_ = 3.165, *p* = 0.04] that did not resist adjustment with PANSS-positive symptoms additionally but was trending toward significance [*F*_(2,738)_ = 2.81, *p* = 0.06] (Table 2). On *post hoc* pairwise analysis, current smokers had lower Kyn levels when compared with past smokers (*p* = 0.04) but after adjusting for PANSS-positive symptoms, this difference was trending toward significance (*p* = 0.05). There were no differences between current smokers and non-smokers (*p* = 0.4) or past smokers and non-smokers (*p* = 0.4).

Kyn:Trp ratio differed among the three groups after adjusting for age, sex, BMI, CPZ equivalent, and PANSS-positive symptoms [*F*_(2,738)_ = 3.61, *p* = 0.03]. On *post hoc* analysis, current smokers had higher Kyn:Trp ratio (*p* = 0.02) when compared with past smokers, but there were no differences between current smokers and non-smokers (*p* = 0.50) or past smokers and non-smokers (*p* = 0.30). There were no differences among the three groups in terms of Trp levels [*F*_(2,738)_ = 0.412, *p* = 0.66].

Comparison of smoking (after consolidating current smokers and past smokers into one group) to non-smoking did not reveal any differences with regards to Kyn (*p* = 0.50), Trp levels (*p* = 0.60), or Kyn:Trp ratio (*p* = 0.70).

### Symptom Severity and Phe, Tyr, and Phe:Tyr Ratio

Positive and negative syndrome scale general psychopathology subscale scores negatively correlated with Phe:Tyr ratio (*n* = 837, coefficient = −4.3, *p* = 0.01) after controlling for age, sex, and BMI but did not show any significant correlation with Phe (*p* = 0.07), Tyr (*p* = 0.4), Kyn (*p* = 0.90), Trp (*p* = 0.90), or Kyn:Trp ratio (*p* = 0.80).

## Discussion

In the first study of its kind, we provide evidence that, among patients with a diagnosis of schizophrenia, smoking is associated with a decrease in Tyr levels. We had previously reported that plasma Phe levels and Phe:Tyr ratio were elevated in schizophrenia patients when compared with normal controls with no difference in continuous Tyr levels. However, having a diagnosis of schizophrenia was associated with Tyr levels in the lowest 25th percentile when compared with healthy controls (40). Our study suggests further differences in Tyr levels within this sample of patients based on their smoking status. As a limitation, we did not have data on smoking status in healthy controls that were included for the parent study and, as such, they could not be used in the current analysis.

Previous work affirmed that smoking induces a Th1 to Th2 shift in immune activation (46). This is consistent with our finding of decreased Kyn levels in current smokers, further aligned with previous reports on decreases in Th1 immunity with reduction in activity of IDO (32). However, Tyr levels decreased, rather than increased, with smoking. This effect may be mediated, independent of inflammation, by nicotine’s ability to increase *TH* gene expression, both centrally and peripherally, thus contributing to the decreased level of Tyr (34, 36, 47).

Finding an elevated Kyn:Trp ratio in current smokers relative to past smokers in our study is not consistent with previously described decreased Kyn:Trp ratio in smokers, which was explained as a consequence of decreased activity of IDO as a result of a putative smoking-induced Th1 to Th2 shift (32). Additionally, finding a high Kyn:Trp ratio and low, rather than high Kyn levels, seems difficult to explain. Nevertheless, this is possible in the event of non-significant decreases in Trp levels due to decreased dietary intake (broadly reported in smokers), increased utilization *via* Trp 2,3-dioxygenase (TDO) pathway [potentially as a result of hypothalamic–pituitary–adrenal (HPA) axis activation as a result of increased vulnerability to stress] or increased availability of vitamin cofactors involved in Kyn metabolism (48).

Because decreased Tyr levels (even after adjustment for demographic variables, BMI, and symptoms scores and reduced to a low grade statistical trend after adjustment for CPZ equivalent) were found in smokers (current and past smokers), it is possible that decreased Tyr levels could be a trait marker for smoking or even contribute to vulnerability for smoking. Patients with schizophrenia have a higher prevalence of smoking when compared with patients with other major mental illnesses (49). The increased prevalence of smoking may not, as some believe, be a consequence of psychosis or progression of the illness, or attempts to achieve symptomatic control, as high rates of smoking in patients with schizophrenia were documented even before their first psychotic episode (50) with the caveat of not excluding the possibility of a link between smoking and prodromal symptoms of the illness. As low Tyr levels are associated with schizophrenia (51), it might be possible that reduced Tyr levels, susceptibility for smoking, and schizophrenia are linked by upstream molecular factors, such as genetic or epigenetic factors. Studies linking allelic variation in the *TH* gene and susceptibility to smoking lends additional credence to this hypothesis (38, 39) within the general context of elevated rates of smoking in first-degree relatives of patients with schizophrenia (52) and higher rates of smoking in twins discordant for the illness (53).

The cross-sectional design of the study is one of the major limitations of this study as it leads to an inability to estimate a direction of causality. Measurement of inflammatory markers like cytokines and C-reactive protein (CRP) would have been useful to additionally assess immune links (e.g., Th1 vs. Th2) but unavailability of sufficient amounts of plasma to assay these markers was prohibitive. We depended on patient reports to determine smoking status as opposed to more objective measures of smoking like serum concentrations of cotinine or carbon monoxide in expired air.

Considering elements of feasibility for achieving the main aims of the parent project, fasting protocols were not observed prior to obtaining blood for determination of plasma amino acid levels, possibly resulting in increased heterogeneity and lower power to uncover significant associations, but there is no reason to believe that a systematic bias spuriously led to our positive results. Food preferences and timing of meals are potential sources of bias as proximity to meals and dietary preference can significantly affect plasma Tyr levels (54). Moreover, smoking has a suppressive effect on appetite, which may independently have interactions with levels of plasma amino acids (55). Furthermore, there are diurnal variations in plasma amino acid levels that have not been taken into account (54). Importantly, plasma levels of amino acids may not accurately represent central availability. We were unable to obtain cerebrospinal fluid (CSF) samples to ascertain amino acid levels, and we do believe that the CSF findings, although theoretically more illuminating, would have little practical value. We have not accounted for the duration of illness, number of past active episodes, or clinical status at the time of study.

We did not have systematic information on potential nicotine supplementation, particularly in past smokers, and, previously, sustained elevations of TH activity after nicotine administration (37) have been documented. We also have not accounted for menstrual status and menstrual cycle phase, which might alter smoking habits and relapses after abstinence (56, 57).

Despite these limitations, the study has several strengths including a large sample size and a relatively homogenous sample of patients with schizophrenia as confirmed by SCID, while excluding other psychosis-spectrum diagnoses like schizoaffective disorder and schizophreniform disorder.

A growing trend in clinical neuroscience is to affirm that only brain (and CSF) biochemistry is truly relevant to psychopathology, i.e., peripheral molecules in the blood are of little relevance for advancing our understanding of mental illness and its manifestations. While, of course, we do not dispute the paramount importance of chemical processes within the brain, we strongly believe that discarding the relevance of blood levels is counterproductive, especially when we refer to precursors of neurotransmitters that can be easily measured in most of the patients. Moreover, levels of certain molecules in blood are predictively associated not only with the metabolism of the brain but also with its functional anatomy. In support of our opinion, several of us have coauthored a recent article reporting that molecules of the Kyn pathway are associated with white matter biochemistry and microstructure (58). Specifically, fasting Kyn:Trp ratio, which was higher in patients with schizophrenia than controls, positively correlated with frontal white matter glutamate levels in both groups. Moreover, in patients, but not in controls, plasma Trp levels correlated negatively with diffusion tensor imaging (DTI) fractional anisotropy (FI), a measure of white matter integrity (58).

Studies with improved methodologies like objective measures of smoking, longitudinal designs, and interventional approaches can elucidate the role that low Tyr plays in relationship to smoking in schizophrenia and if there is any potential of uncovering new targets for pharmacological interventions. These efforts are well justified considering the staggering rates of smoking and increased cardiovascular mortality in schizophrenia patients.

## Author Contributions

Drs. TTP and DR contributed equally and share senior authorship. TTP and DR designed the study. TTP and DR secured funding, with later contribution from AJM. DR led the data collection. IG, AMH, BK, AJM, JK, MF, and DR took part in data collection and initial data management. OO performed the secondary data management. DF performed the biochemical analysis and contributed to interpretation of results. AJM, JK, OO, DR, and TTP were involved in data analysis and interpretation. GMR, CAL, XP, CG, MWG, and RNR provided critical intellectual input to reconcile diverging results, analyzing and integrating alternative interpretations. CAL, TTP and RNR particularly contributed to interpretation of the finding from a neuroscience standpoint; GMR, MWG, CG, JK and OO from a clinical relevance standpoint. JK and AJM wrote the first two drafts of the manuscript with input from OO and TTP. CAL, CG, TTP, RNR, OO, and DF provided expert editing and reorganization of the manuscript. All authors contributed to editing the final version of the manuscript.

## Conflict of Interest Statement

The authors declare that the research was conducted in the absence of any commercial or financial relationships that could be construed as a potential conflict of interest.
